# Supplementary material for: A lipid-binding protein mediates rhoptry discharge and invasion in Plasmodium falciparum and Toxoplasma gondii parasites
Source: Nat Commun. 2019 Sep 6;10:4041. doi: 10.1038/s41467-019-11979-z (PMC6731292; doi:10.1038/s41467-019-11979-z)
Supplement: Supplementary file 1 — Supplementary Information [file 41467_2019_11979_MOESM1_ESM.pdf]

Supplementary information for

A lipid-binding protein mediates rhoptry discharge and invasion in *Plasmodium falciparum* and *Toxoplasma gondii* parasites

Catherine Suarez[1], Gaëlle Lentini[1&], Raghavendran Ramaswamy[2&], Marjorie Maynadier[1&], Eleonora Aquilini[1], Laurence Berry-Sterkers[1], Michael Cipriano[3], Allan Chen[4], Peter Bradley[4], Boris Striepen[3], Martin J. Boulanger[2], Maryse Lebrun[1]*

correspondence to: maryse.lebrun@umontpellier.fr

This file includes:

Supplementary Figures 1 to 12

Supplementary Tables 1 to 2

Supplementary Notes 1 to 3

Supplementary References

Supplementary Figures

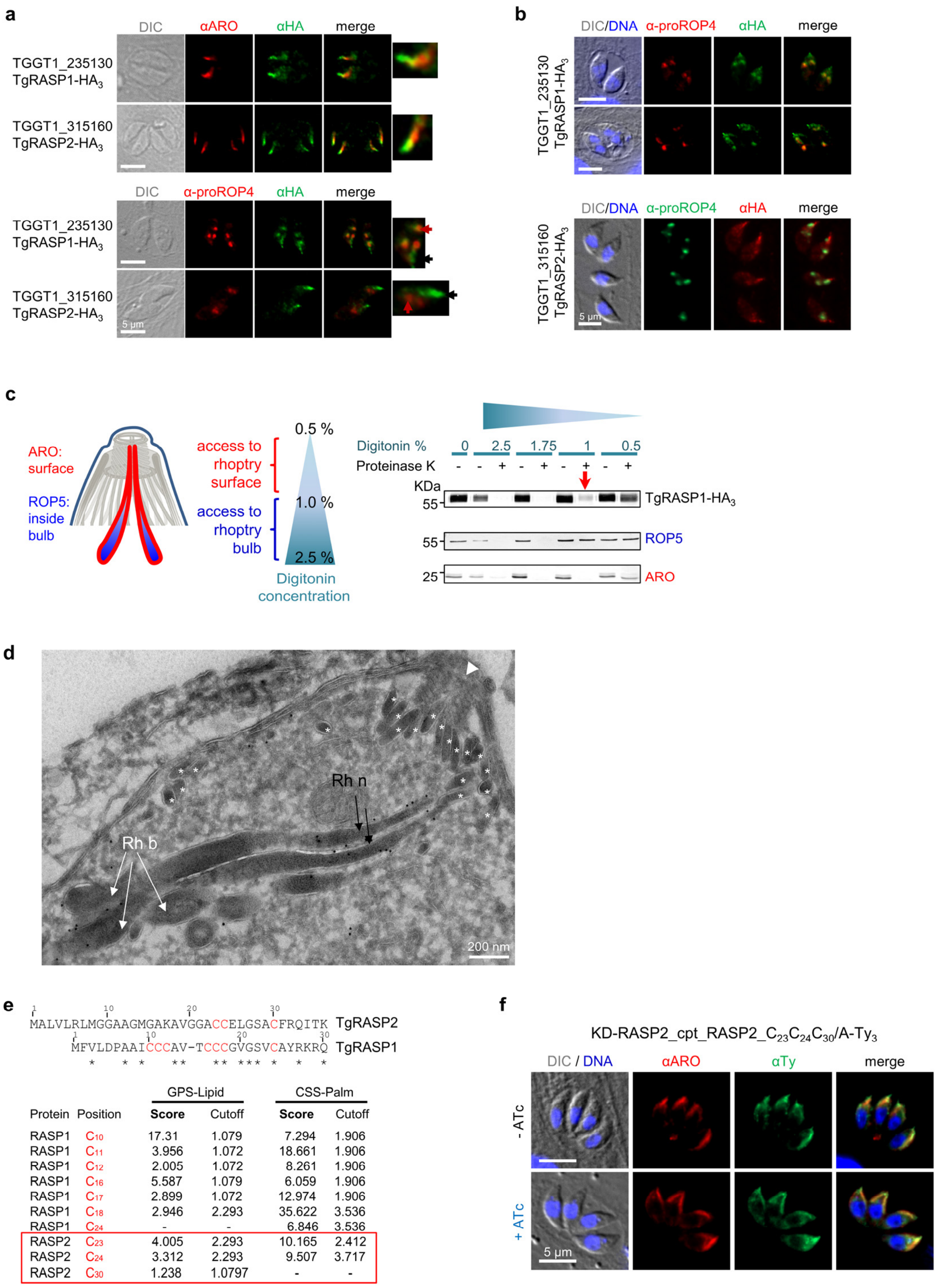

		GPS-Lipid		CSS-Palm	
Protein	Position	Score	Cutoff	Score	Cutoff
RASP1	C_{10}	17.31	1.079	7.294	1.906
RASP1	C_{11}	3.956	1.072	18.661	1.906
RASP1	C_{12}	2.005	1.072	8.261	1.906
RASP1	C_{16}	5.587	1.079	6.059	1.906
RASP1	C_{17}	2.899	1.072	12.974	1.906
RASP1	C_{18}	2.946	2.293	35.622	3.536
RASP1	C_{24}	-	-	6.846	3.536
RASP2	C_{23}	4.005	2.293	10.165	2.412
RASP2	C_{24}	3.312	2.293	9.507	3.717
RASP2	C_{30}	1.238	1.0797	-	-

Supplementary Figure 1 │ Expression and localization of RASP proteins

a, b, Immunofluorescence assay (IFA) of HA-tagged TGGT1_235130 (TgRASP1) and TGGT1_315160 (TgRASP2) tachyzoites. Individual panels of Fig. 1a are shown in panel

b. **c**, Proteinase K digestion assay. Left: schematic representation of the permeabilization assay. Right: Immunoblot showing differential accessibility of proteinase K depending on the digitonin concentration used to permeabilize TgRASP1-HA_3 parasites. Blots were incubated with rat anti-HA, mouse anti-ROP5 and rabbit anti-ARO antibodies. **d**, Immuno-electron microscopy of TgRASP2. Section of two full-length rhoptries. Conoid (white triangle), rhoptry neck (Rh n; black arrow), rhoptry bulb (Rh b; white arrows) and micronemes (white asterisks). **e**, Prediction for palmitoylation sites in TgRASP1 and TgRASP2 proteins. Upper part: alignment using Geneious (https://www.geneious.com) of the N-terminal ends of TgRASP1 and TgRASP2 proteins. Conserved residues are indicated with an asterisk. Lower part: the cysteine residues predicted to be palmitoylated using GPS-lipid2 and CSS-Palm3 are in red. Red frame indicates the residues that were mutated. **f**, IFA using anti-Ty antibodies on the KD_RASP2 complemented with a mutated version of TgRASP2 at the predicted palmitoylation sites. The mutations converted the cysteines C23, C24 and C30 to alanines. Source data are provided as a Source Data file.

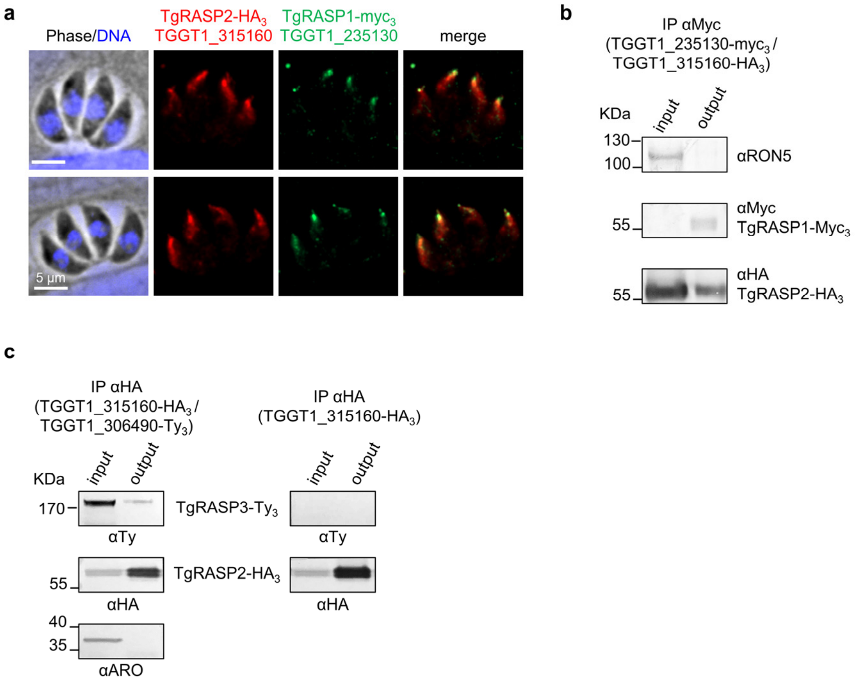

Supplementary Figure 2 │ Interactions between TgRASP proteins.

a, IFA of TgRASP1-myc3 and TgRASP2-HA3 parasites using anti-Myc and anti-HA antibodies reveals co-localization of the two proteins. **b**, Co-immunoprecipitation using anti-c-Myc antibodies on parasites expressing TgRASP1-myc3 and TgRASP2-HA3, followed by immunoblots using anti-Myc and anti-HA antibodies show the interaction between TgRASP1 and TgRASP2. The rhoptry neck protein RON5[1] is used as negative control to show the specificity of the interaction. **c**, Left: co-immunoprecipitation using anti-HA antibodies on parasites expressing TgRASP2-HA3 and TgRASP3-Ty3 followed by immunoblots using anti-Ty and anti-HA antibodies shows the interaction between TgRASP2 and TgRASP3. ARO is used as negative control to show the specificity of the interaction[2]. Right: control immunoprecipitation using anti-HA antibodies on parasites expressing only TgRASP2-HA3. Source data are provided as a Source Data file.

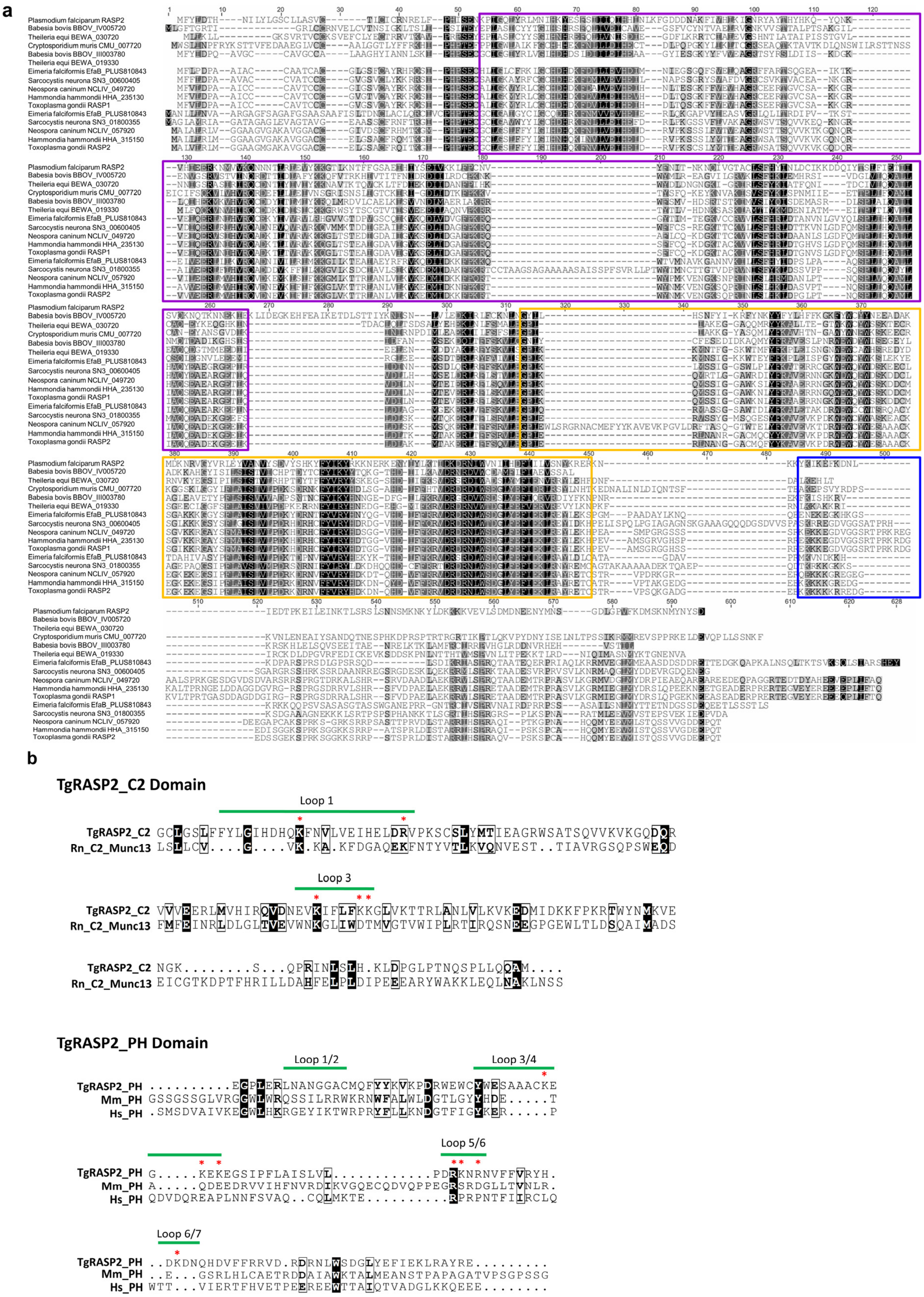
a
Plasmodium falciparum RASP2
Babesia bovis BBOV_IV005720
Theileria equi BEWA_030720
Cryptosporidium muris CMU_007720
Babesia bovis BBOV_III003780
Theileria equi BEWA_019330
Eimeria falciformis EfaB_PLUS810843
Sarcocystis neurona SN3_00600405
Neospora caninum NCLIV_049720
Hammondia hammondii HHA_235130
Toxoplasma gondii RASP1
Eimeria falciformis EfaB_PLUS810843
Sarcocystis neurona SN3_01800355
Neospora caninum NCLIV_057920
Hammondia hammondii HHA_315150
Toxoplasma gondii RASP2
b
TgRASP2_C2 Domain
Loop 1
TgRASP2_C2 GCLGSLFFYLGIHDHQKFNVLVEIHELDRVPKSCSLYMTIEAGRWSATSQVVKVKGQDQR
Rn_C2_Munc13 LSLLCV....G....VK.KA.KFDGAQEKFNTYVTLKVQNVEST..TIAVRGSQPSWEQD
Loop 3
TgRASP2_C2 VVVEERLMVHIRQVDNEVKIFLFKKGLVKTTRLANLVLKVKEDMIDKKFPKRTWYNMKVE
Rn_C2_Munc13 FMFEINRLDLGLTVEVWNKGLIWDTMVGTVWIPLRTIRQSNEEGPGEWLTLDSQAIMADS
TgRASP2_C2 NGK........S...QPRINLSLH.KLDPGLPTNQSPLLQQAM....
Rn_C2_Munc13 EICGTKDPTFHRILLDAHFELPLDIPEEEARYWAKKLEQLNAKLNSS
TgRASP2_PH Domain
Loop 1/2
Loop 3/4
TgRASP2_PH ..........EGPLERLNANGGACMQFYYKVKPDRWEWCYWESAAACKE
Mm_PH GSSGSSGLVRGGWLWRQSSILRRWKRNWFALWLDGTLGYYHDE.....T
Hs_PH .SMSDVAIVKEGWLHKRGEYIKTWRPRYFLLKNDGTFIGYKER.....P
Loop 5/6
TgRASP2_PH G.....KEKEGSIPFLAISLVL............PDRKNRNVFFVRYH.
Mm_PH A.....QDEEDRVVIHFNVRDIKVGQECQDVQPPEGRSRDGLLTVNLR.
Hs_PH QDVDQREAPLNNFSVAQ..CQLMKTE..........RPRPNTFIIRCLQ
Loop 6/7
TgRASP2_PH ..DKDNQHDVFFRRVD.RDRNLWSDGLYEFIEKLRAYRE.........
Mm_PH ..E...GSRLHLCAETRDDAIAWKTALMEANSTPAPAGATVPSGPSSG
Hs_PH WTT...VIERTFHVETPEEREEWTTAIQTVADGLKKQEEE........

Supplementary Figure 3 │ Multiple sequence alignment of TgRASP2 homologues in Apicomplexa and alignment of TgRASP2 putative C2 and PH domains with mammal C2 and PH domains.

a, BLAST analysis of the TgRASP2 protein sequence revealed orthologues in Apicomplexa. The C2 and PH domains identified using Phyre2 are boxed in purple and yellow respectively. The region containing polybasic residues is boxed in blue. **b**, Structure based sequence alignment of TgRASP2_C2 and TgRASP2_PH domains. The top hits predicted by Phyre2[3] and ITASSER[4-6] were used for the alignment. Only one sequence was used for the alignment in the case of TgRASP2_C2 as other hits had very low sequence identity (<12 %) and their inclusion resulted in an alignment that was below a statistical threshold of significance. The loops that may be involved in lipid binding are indicated in green. The nomenclature of the loops is the same as described in Supplementary Fig. 9. The red asterisks indicate the residues that were mutated to Asp in the recombinant protein TgRASP2con2^{MUT}. The unshaded boxes indicate similar amino acids whereas the shaded/black boxes indicate conserved residues. The accession number and PDB ID of sequences are: TgRASP2- *Toxoplasma gondii* RASP2 (ToxoDB- TGME49_315160), Rn_C2_Munc13- *Rattus noverigus* C2 Munc 13(PDB id- 2CJS), Mm_PH- *Mus musculus* PH (PDB id- 2D9V), Hs_PH- *Homo sapien* PH (PDB id- 1UNQ).

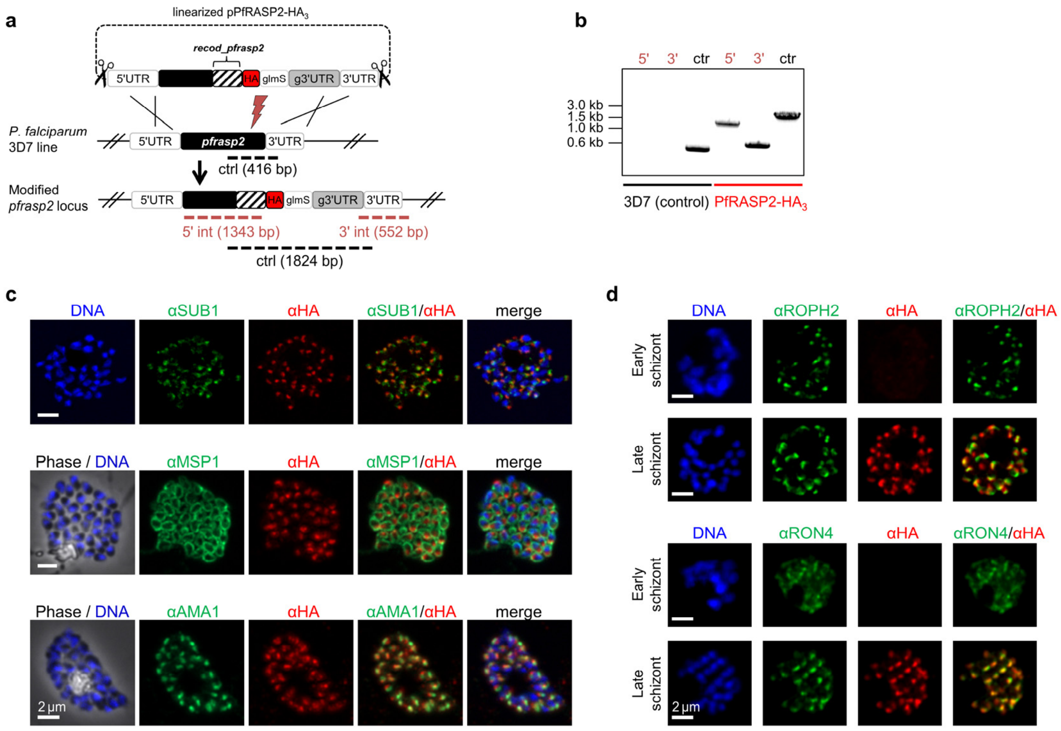

Supplementary Figure 4 | Epitope tagging of PfRASP2 and expression in late erythrocytic stages.

a, Schematic representation depicting the double crossover homologous recombination strategy to epitope-tag PfRASP2 in the *P. falciparum* 3D7 line. **b**, Integration PCRs on the PfRASP2-HA3 clonal line and the 3D7 (control) performed with primers 2910/2911 (5' integration), 2908/2909 (3' integration) and 2912/2913 to control the locus (wt = 416 bp; modified locus = 1824 bp). **c**, IFAs on PfRASP2-HA3 schizonts showing PfRASP2 with different late stage markers; the exoneme marker PfSUB1, the merozoite surface protein PfMSP1, and the micronemal protein PfAMA1. **d**, IFAs of HA-tagged PfRASP2 parasites. Individual panels of Fig. 3a are shown here. Source data are provided as a Source Data file.

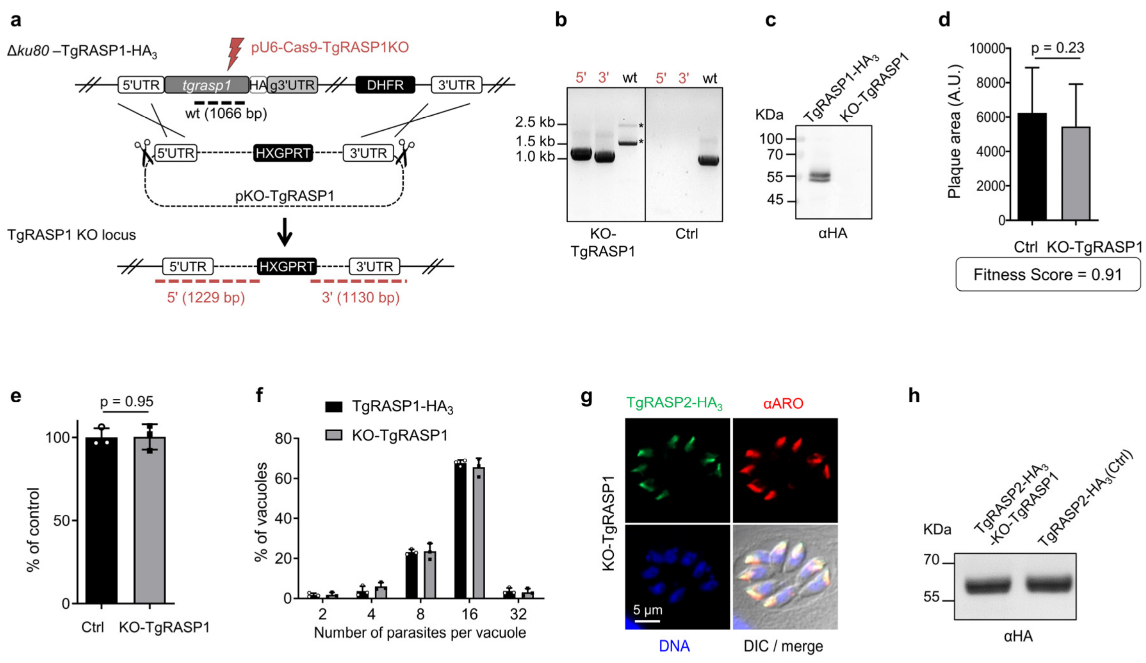

Supplementary Figure 5│ TgRASP1 is dispensable for the tachyzoite lytic cycle.

a, Scheme illustrating the approach used to remove *tgrasp1* in *Δku80* TgRASP1-HA3 parasites using CRISPR/Cas9 nuclease mediated gene recombination strategy. **b**, Integration PCRs showing double homologous recombination of the HXGPRT gene at the TgRASP1 locus. 5' integrations PCR performed with primers 1377/1042, 3' integration PCR with primers 1589/1043 and PCR on the wt locus with primers 1156/1475. *Asterisks = unspecific amplifications. **c**, Immunoblot using anti-HA antibodies on TgRASP1-HA3 and KO-TgRASP1 parasites. d, Plaque assay of KO-TgRASP1 vs TgRASP1-HA3 parasites on HFF monolayers after 7 days. Lysis plaque areas were measured in three independent experiments. AU: arbitrary units. Mean value of plaque area ± SD (n=38 for TgRASP1-HA3, n= 26 for KO-TGRASP1) for one representative experiment. CRISPR fitness score was derived from Ref.[7]. **e**, 5 min invasion assay of Δ*ku80* TgRASP1-HA3 (Ctrl) and KO-TgRASP1 shows no defect in host cell entry for TgRASP1-depleted parasites. (D, E) Values represent means ± SD, n=3, from a representative experiment out of 3 independent

assays. **f**, Intracellular replication of TgRASP1-HA3 (Ctrl) and KO-TgRASP1 parasite lines. The percentage of vacuoles containing 2, 4, 8, 16 or 32 parasites was determined on 200 vacuoles. Values represent means ± SD, n=3, from a representative experiment out of 3 independent assays. **g**, IFA using anti-HA antibodies on KO-TgRASP1 parasites expressing TgRASP2-HA3 shows correct localisation of TgRASP2 at the rhoptries in the absence of TgRASP1. **h**, Immunoblot showing the same expression level of TgRASP2 in control (TgRASP2-HA3) and KO-TgRASP1 parasites. TgRASP1 deletion does not affect expression and localization of TgRASP2. Source data are provided as a Source Data file.

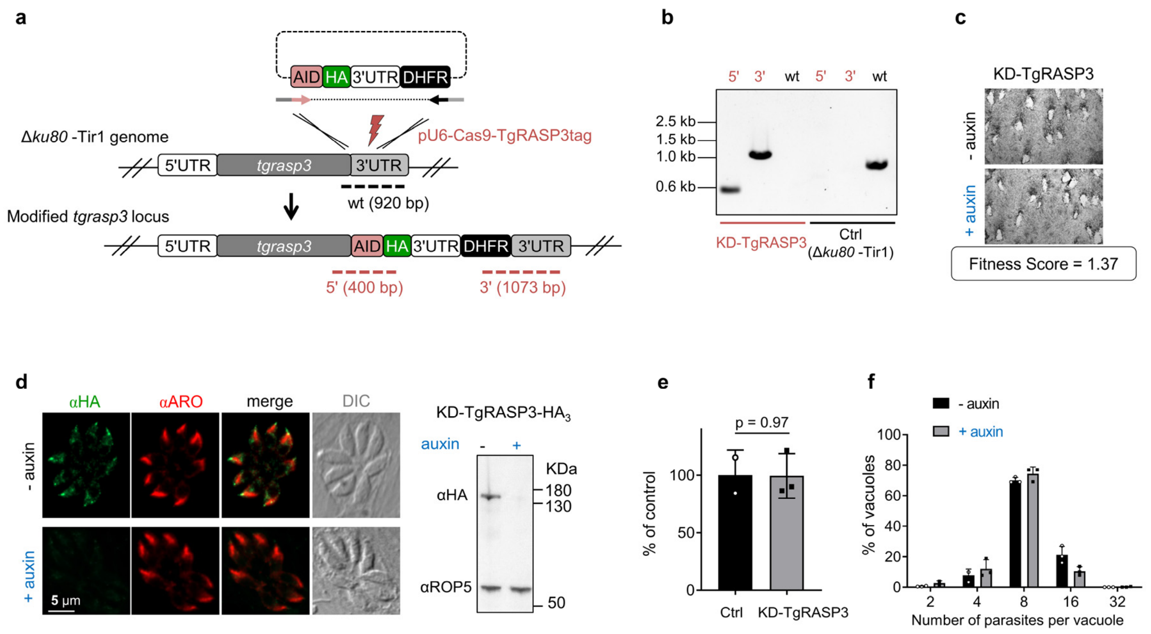

Supplementary Figure 6 | TgRASP3 is dispensable for the tachyzoite lytic cycle.

a, Schematic representation depicting the approach used to integrate a triple HA tag and the auxin-inducible degron (AID) to TgRASP3 in *Δku80* parasites using CRISPR/Cas9 nuclease mediated gene recombination strategy. **b**, Integration PCRs showing double homologous recombination of the AID integration at the TgRASP3 locus. 5' integrations PCR performed with primers 2969/1476, 3'integration PCR with primers 3192/2601 and PCR on the wt locus with primers 2969/3192. **c**, Plaque assay of KD-TgRASP3 ± IAA on HFF monolayers after 6 days. One representative experiment out of three independent assays. CRISPR fitness score was derived from Ref.[7]. **d**, Left: IFA on KD-TgRASP3 -HA3 ± 24 h IAA parasites shows correct localization of the rhoptries (TgARO) in the absence of TgRASP3. Right: Immunoblot using anti-HA antibodies showing depletion of TgRASP3 upon auxin treatment of KD-TgRASP3-HA3 parasites. ROP5, loading control. **e**, 5 min invasion assay of control and KD-TgRASP3 +48 h IAA shows no defect in host cell entry for TgRASP3-depleted parasites. **f**, Intracellular replication of KD-TgRASP3 parasites ± 48 h IAA. The percentage of vacuoles containing 2, 4, 8, 16 or 32 parasites was

determined on 200 vacuoles. (C, E, F) Values represent means ± SD, n=3, from one experiment. Source data are provided as a Source Data file.

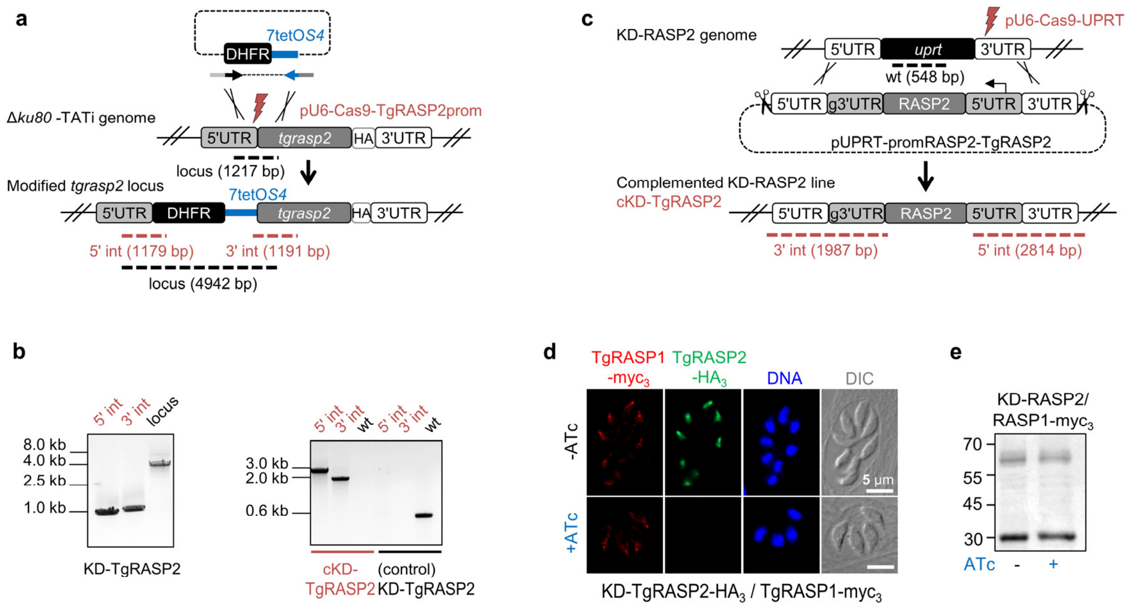

Supplementary Figure 7 | Depletion of RASP2 in *Toxoplasma* does not affect replication, egress, and gliding motility.

a, Scheme illustrating the approach used to replace the endogenous promoter of TgRASP2 by a tetracycline repressible promoter (7tetOS4). TATi_TgRASP2-HA_3 parasites were modified using the CRISPR/Cas9 nuclease mediated gene recombination system. **b**, Left: integration PCRs of the 7TetO inducible promoter at the *tgrasp2* locus. 5' integrations PCR performed with primers 2455/2454, 3' integration PCR with primers 2456/1810 and PCR on the locus (1217 or 4942 bp) with primers 2455/1778**.** Right: PCRs showing integration of the TgRASP2 gene at the UPRT locus in the complemented clones D7 and B8; 5' integrations PCR performed with primers 1431/2419, 3' integration PCR with primers 2906/1428 and control PCR on the wt locus with primers 1432/1433. **c**, Strategy used to complement the KD-TgRASP2 line. An additional copy of *tgrasp2* driven by its own promoter was integrated at the UPRT locus by double homologous recombination. **d**, IFA on KD-TgRASP2-HA_3/TgRASP1-Myc_3 ± ATc 48 h shows correct localisation of TgRASP1 to the rhoptries in the absence of TgRASP2. **e**, Immunoblot showing the same

expression level of TgRASP1 in control and TgRASP2-depleted parasites. TgRASP2 deletion does not affect expression and location of TgRASP1. Source data are provided as a Source Data file.

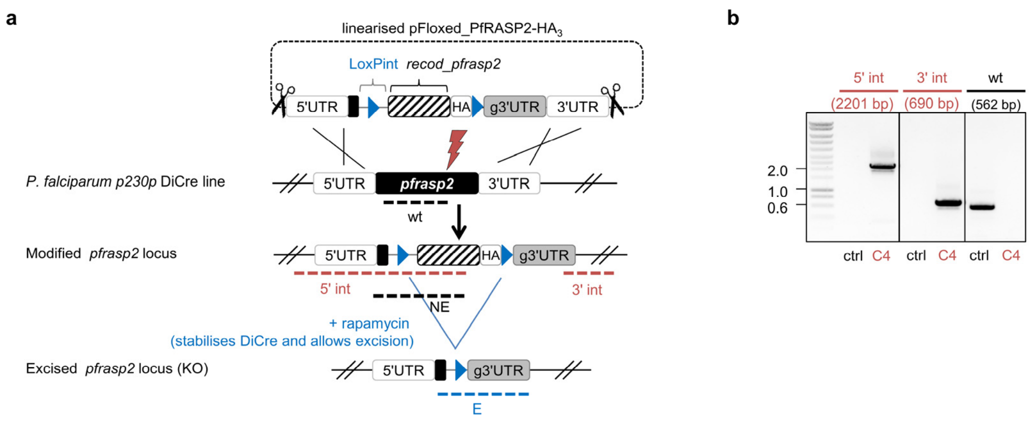

Supplementary Figure 8 | Strategy to conditionally excise *pfrasp2*.

a, Schematic representation depicting the double crossover homologous recombination strategy to simultaneously flox and epitope-tag the *pfrasp2* gene in the 3D7 DiCre *p230p* line. **b**, Integration PCRs on the clonal line iKO-PfRASP2-HA3 (clone C4) and the 3D7 DiCre *p230p* (ctrl) performed with primers 3203/2911 for 5' integration, 2881/2882 for 3' integration and 2792/2749 for the unmodified wt locus. Source data are provided as a Source Data file.

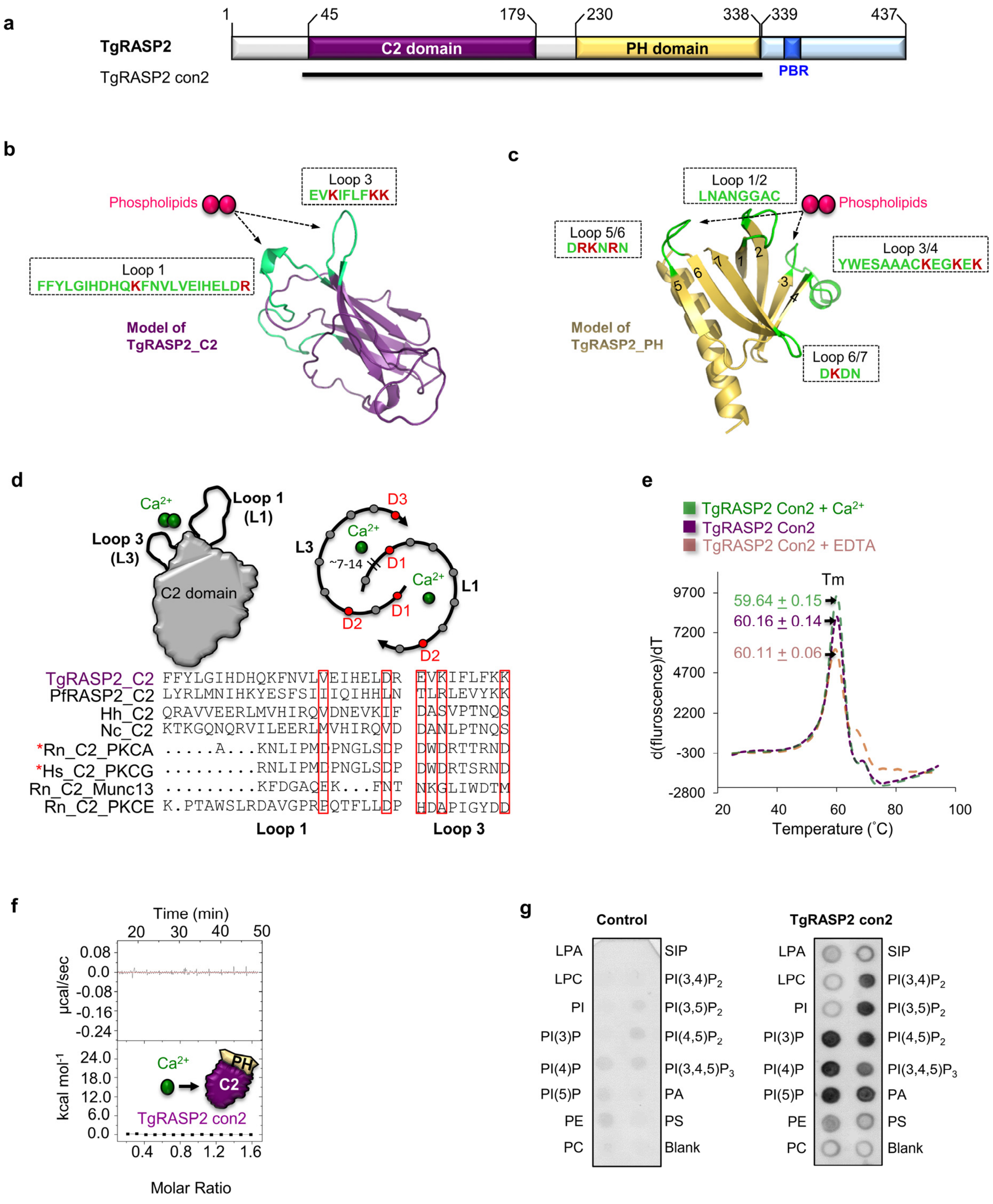

Supplementary Figure 9 │ TgRASP2 does not appear to bind Ca^{2+}

a, Schematic representation of TgRASP2 highlighting domain boundary predictions and expression construct of TgRASP2con2. PBR- polybasic residues that extend from residues 354-362. **b** and **c**, Homology modelling of TgRASP2_C2 (Gly45–Leu179) and PH

(Glu230-Glu338) with phospholipids (pink spheres) binding loops indicated in green and the potential phospholipid residues highlighted in red. **d**, Schematic representation showing the roles of loops 1 and 3 in Ca^{2+} coordination by canonical C2 domains. The number of aspartate residues that coordinate calcium in each loop are indicated. Loop 1 is typically 10-20 amino acids long. Bottom: Comparison of the putative C2 domains from apicomplexan TgRASP2 homologs (*Toxoplasma gondii* C2 (TgRASP2_C2)- ToxoDB-TGME49_315160 ; *Plasmodium falciparum* RASP2 C2 (PfRASP2_C2)- PlasmoDB-PF3D7_0210600; *Hammondia hammondi* C2 (Hh_C2)- NCBI ref- XP_008883432.1; *Neospora caninum* C2 (Nc_C2) NCBI reference- XP_003885397.1) and sequences of Ca^{2+} dependent and independent C2 domains (*Rattus norvegicus* C2 domain of protein kinase C alpha (Rn_C2_PKCA)- PDB id- 3GPE; *Homo sapiens* C2 domain of protein kinase C gamma (Hs_C2_PKCG)- PDB id- 2UZP; *Rattus norvegicus* C2 Munc 13(Rn_C2_Munc13)- PDB id- 2CJS; *Rattus norvegicus* C2 domain of protein kinase C epsilon (Rn_C2_PKCE)- PDB id- 1GMI)). The red asterisk indicates the Ca^{2+} binding C2 domains and the red boxes indicate the Ca^{2+} coordinating residues in loop 1 and 3. **e**, Comparison of the unfolding transitions of native TgRASP2con2 (deep purple) following EDTA (orange) and Ca^{2+} (green) treatments. The melting temperature (Tm) values for the curves are indicated with their standard deviations. Each value represents a single experiment with 3 replicates. **f**, Representative ITC binding isotherm following the titration of $CaCl_2$ into a solution of TgRASP2con2. **g**, Lipid blot assay using a control protein and rTgRASP2con2 (same exposure time). LPA, Lysophosphatidic acid. LPC, Lysophosphatidic Acid. PI, Phosphoinositol. PI3P, Phosphoinositol-3-phosphate. PI4P, Phosphoinositol-4-phosphate. PI5P, Phosphoinositol-5-phosphate. PE, Phosphatidylethanolamine. PC, Phosphatidylcholine. SIP, Shingosine-1-phosphate.

PI(3,4)P_2, Phosphatidylinositol-3,4-biphosphate. PI(3,5)P_2, Phosphatidylinositol-3,5-biphosphate. PI(4,5)P_2, Phosphatidylinositol-4,5-biphosphate. PI(3,4,5)P_3, Phosphatidylinositol-3,4,5-triphosphate. PA, Phosphatidic acid. PS, Phosphatidylserine. Source data are provided as a Source Data file.

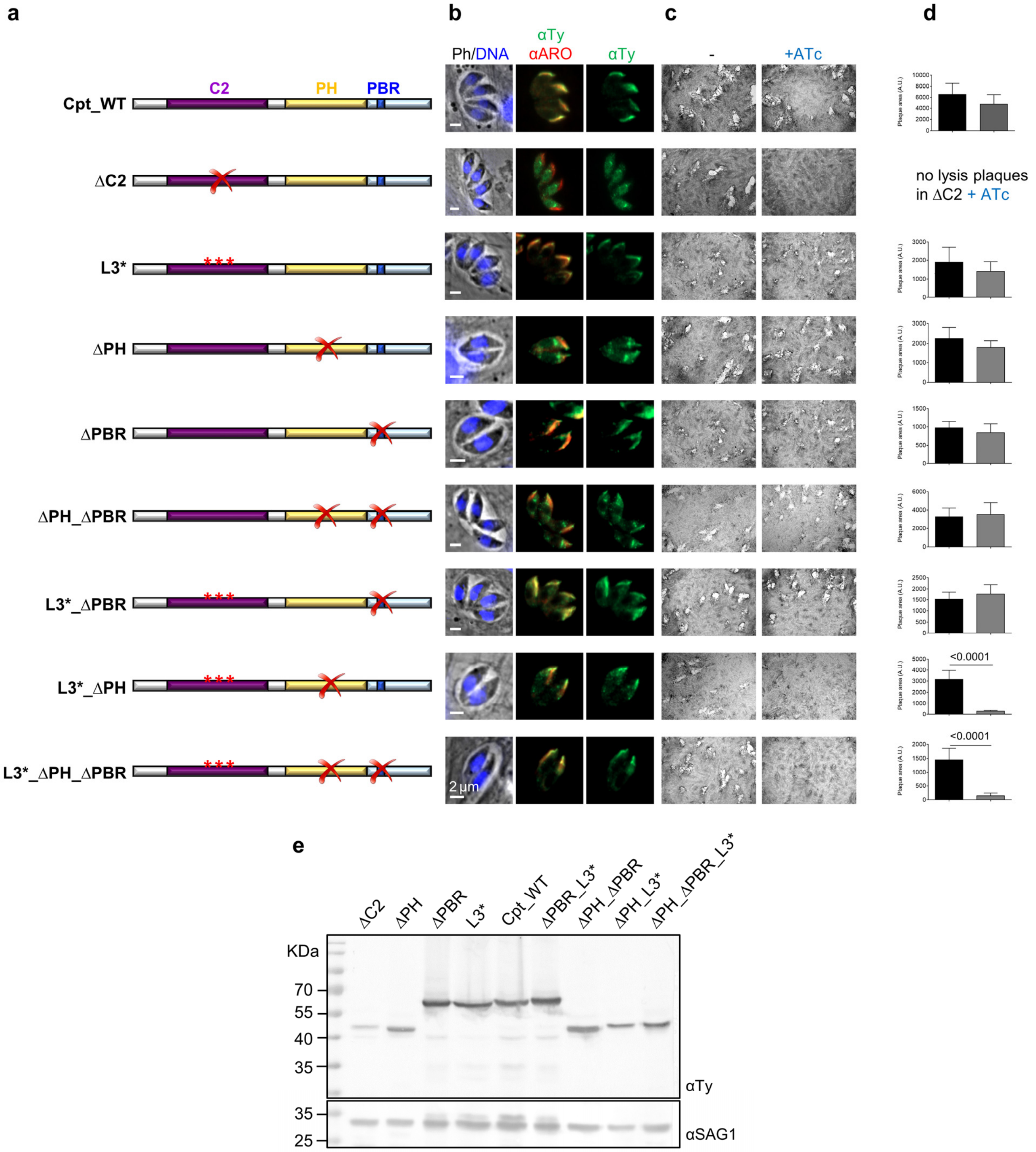

Supplementary Figure 10│ Complementation mutants of TgRASP2 show a cooperative role of the PH domain and three basic residues of loop 3 in the C2 domain. **a**, Schematic representation of TgRASP2 mutants in complemented strains. The parts mutated or deleted are highlighted by red asterisks or crossed out respectively. Cpt_WT,

complementation with wild-type copy of TgRASP2. ΔC2, deletion of AA62 to 179. L3*, triple point mutations of the lysines to aspartic acid residues at positions K123 /K128 /K129 of the C2 loop3. ΔPH, deletion of AA230 to 363. ΔPBR, deletion of the stretch of polybasic residues (AA380 to 387). ΔC2_ΔPH, deletion of the putative C2 and putative PH domains. ΔPH_ΔPBR**,** deletion of the putative PH and PBR domains. L3*_ΔPBR, mutation within the loop3 of the C2 domain and deletion of the stretch of polybasic residues. L3*_ΔPH, mutation within the loop3 of the C2 domain and deletion of the PH domain. L3*_ΔPH_ΔPBR, mutation within loop3 of the C2 domain, deletion of the putative PH domain and deletion of stretch of polybasic residues. **b**, IFAs of the different complemented lines. **c** and **d**, Plaque assays of the different complemented mutants of TgRASP2 on HFF monolayers after 6 days in the absence (black bars) or presence of ATc (grey bars). Lysis plaque areas were measured in ≥ 2 independent experiments. AU: arbitrary units. Mean value of plaque area ± SD (n ≥15 for -ATc, n ≥ 15 for +ATc) for one representative experiment for each mutant. **e**, Immunoblot using anti-Ty antibodies to detect the complementing copies of TgRASP2. SAG1, loading control.

Fig. 1d

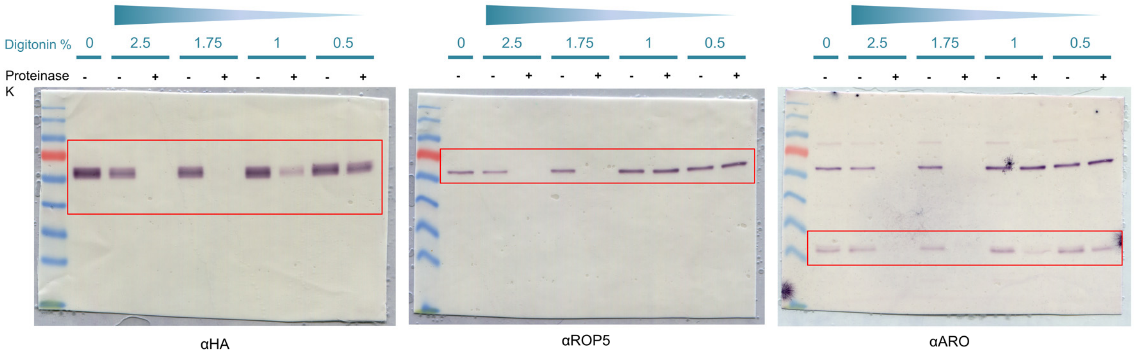

Fig. 4b

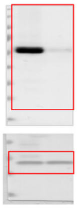

Fig. 5b

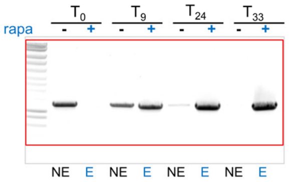

Fig. 5d

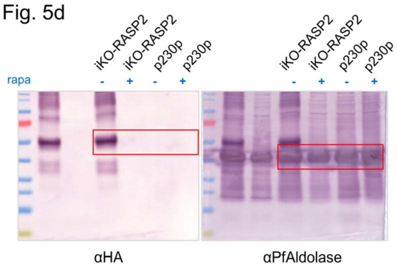

Fig. 6a

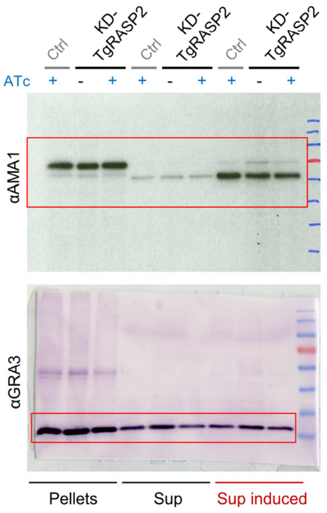

Fig. 6b

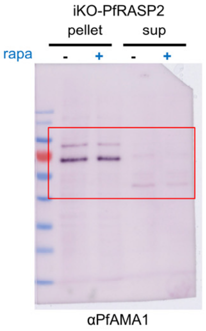

Fig. 7b

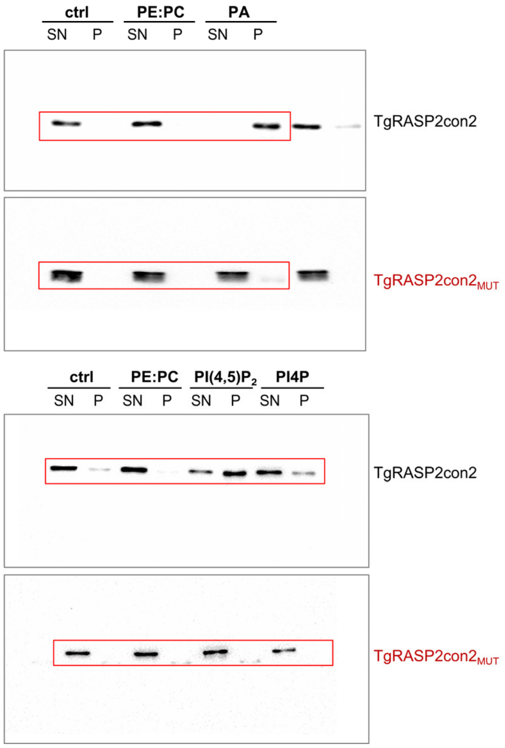

Supplementary Figure 11 | Unedited full-length gels/blots used in main text.

Shown are unmanipulated (raw) versions of the images shown in Figure 1d, Figure 4b, Figure 5b, Figure 5d, Figure 6a-b and Figure 7b. The images have been edited only to include boxes (red) that highlight which parts of the full blots are reproduced in the corresponding figures.

SFig. 1c

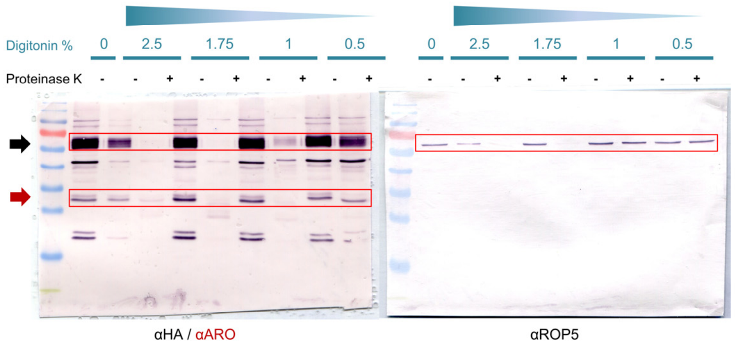

SFig. 2b

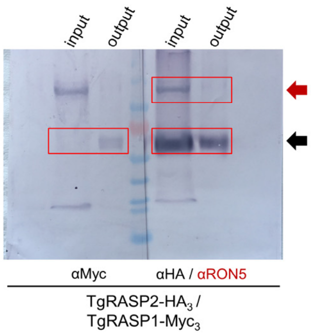

SFig. 2c

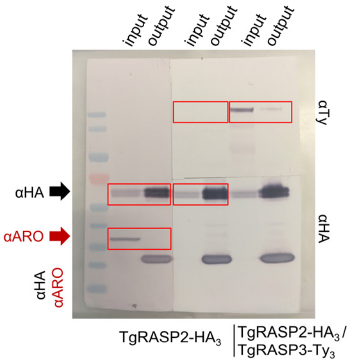

SFig. 4a

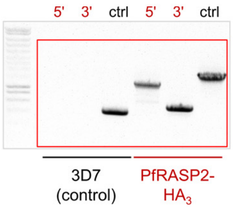

SFig. 5b

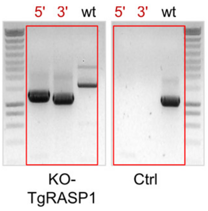

SFig. 5c

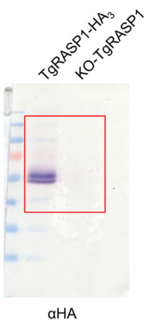

SFig. 5h

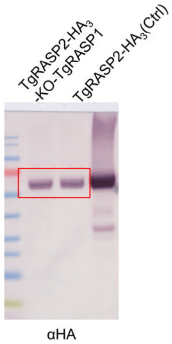

SFig. 6b

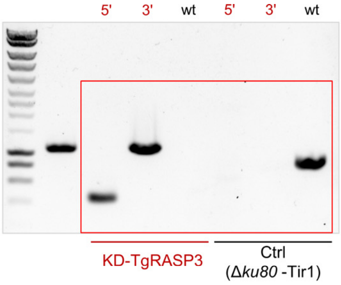

SFig. 6d

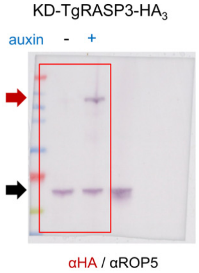

SFig. 7e

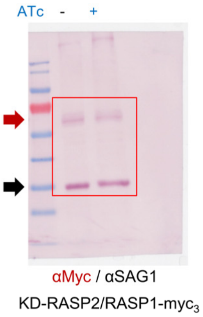

SFig. 7b

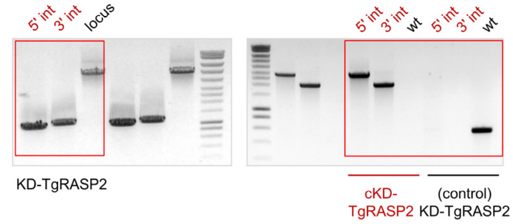

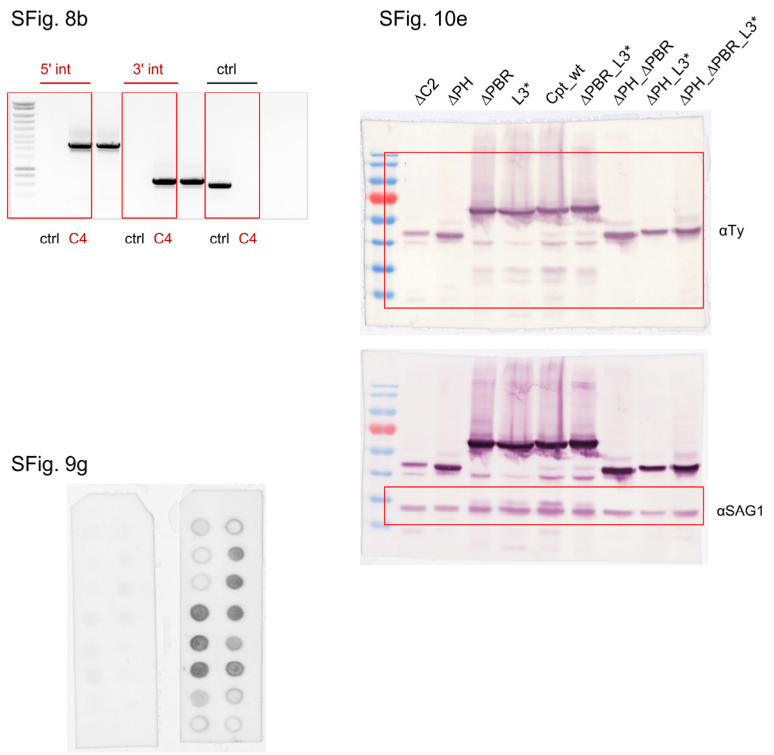

Supplementary Figure 12 | Unedited full-length gels/blots used in Supplementary information figures.

Shown are unmanipulated (raw) versions of the images shown in Supp. fig.1c, Supp. fig.2b-c, Supp. fig.4a, Supp. fig. 5b-c and h, Supp. fig. 6b and d, Supp. fig.7b and e, Supp. fig.8b, Supp. fig.9g, Supp. fig.10e. The images have been edited only to include boxes (red) that highlight which parts of the full gels/blots are reproduced in the corresponding figures.

Supplementary Table 1 │ Y2H screen using TgRASP2 as a bait.

Prey	Clone name	Position start…Stop(n)	Score
**TGGT1_306490**	pB29_A-16	9..1247	**A**
Hypothetical TgRASP3	pB29_A-7	279..1179	
	pB29_A-14	279..1179	
	pB29_A-23	279..1179	
	pB29_A-2	408..1069	
	pB29_A-21	438..1176	
	pB29_A-32	438..1176	
	pB29_A-1	438..1176	
	pB29_A-24	477..986	
**TGGT1_206430**	pB29_A-34	13212..13858	**C**
Formin 1	pB29_A-4	13386..14324	
**TGGT1_319860**	pB29_A-	27600..1568	**D**
DNA polymerase			
**TGGT1_200385**	pB29_A-8	2562..4092	**D**
DNA-binding domain Containing protein	pB29_A-11	2562..4092	
**TGGT1_313910**	pB29_A-6	1788..2322	**D**
RNA recognition motif 2 protein	pB29_A-15	1788..2322	
	pB29_A-12	1788..2322	
	pB29_A-28	1788..2322	
	pB29_A-26	1788..2322	
**TGGT1_279100**	pB29_A-25	447..1790	**D**
MAF1a			
**TGGT1_313340**	pB29_A-5	3483..3666	**D**
Hypothetical			
**TGGT1_212880**	pB29_A-33	13911..14279	**D**
CEP250			

Supplementary Table 2 │ List of primers used.

Prime	5' → 3'
1042	GCCGACAGGACGCTACTGG
1123	ATCTTCCAATCCAATTTAATGCGGTAAGGCTGTGGTGGTTCG
1124	TCCTCCACTTCCAATTTTAGCCTGAGTAAAAAGTAAGGGTTGCTCC
1156	GGCATCGCCGGTGCTTCC
1377	GTCGTCGCCTTCGTCTTC
1418	TACTTCCAATCCAATTTAATGCCCTTCGCCGCGTTG
1419	TCCTCCACTTCCAATTTTAGCCGTTTGAGGTTCTTCGTCTC
1428	ATCTTCCAGCGAACGCTTTC
1431	ATACGTCTTCAGCACACCACC
1432	GAAGAACGACGCTGCAAAC
1433	TTGCCATGTCAAGTTCCTACC
1475	GTGAAACACAGGACGGTCTC
1476	GTACGACGTCCCGGACTACGCTG
1585	TATACTCGAGTTTCTCTCCTCATGCAAGCC
1586	ATAAAGCTTAAAAGGACCGACAATCAGGG
1587	TATAGGATCCGGAAGATGCTCGTCCAACA
1588	TCTAGATGTCCTTGGGCTATTCGAG
1778	TCGAGGCCGTAAAGTACCG
1810	GTTCCCTTCGAGTCCGAA
2066	AAGTTGCGAGGCAGACGAAAGACCAG
2067	AAAACTGGTCTTTCGTCTGCCTCGCA
2087	AAGTTGCTCCCACGTCCCTCACCATG
2088	AAAACATGGTGAGGGACGTGGGAGCA
2418	GTGTTCGATTGCCTCTTCAA
2419	TTGAAGAGGCAATCGAACAC
2420	GTTCTCTGAAGAGGCCTTGTAGTGCACAGGCTAAGCTTCGCCAGGCTGTA
2421	GCCGCCCATCAGGCGCAAGACGAGCGCCATAGATCTGGTTGAAGACAGAC
2454	ACGGGAGGCCGTTGTTGAT
2455	CAGTGGCTGTGTTCCTCTCGT
2456	ACGCGTCGCAGCATACT
2682	GCAGCCTCACGTTAACGCCTCCATGTGCCAGTTGTCTC
2683	AGGCGCCTCCAACAGCTTT
2684	AGCTGTTGGAGGCGCCTGCTGTGAACTGGGAAGCGCATGTTT
2688	AAGTTTTTGTTCAGCGATCGTTTGAGGTTCTTCGTCT
2739	GATGGAATCAGGGGCAGAAG
2742	CATGTTATCAGTAGGCTAACTTCG
2748	GGGATCCCCCCGGGTAGCCAGATATGCCTATACACACTACCAC
2749	ATTTCAAGAATTTCTTTCGGAGTGTCTTCTATTAAATTATCTTTAAATTC
2750	TTTAATAGAAGACACTCCTGAAAGAAATTCTTGAAATAAACAAAACTC
2751	CCATGGTTAAGCGGCAGGCTAGGCATAATCTGGAACATCG

2752	ATCGAGATCTAGTCTGCAGAACTCAGGAGATTTAGGTCCAGTA
2753	TTCTTCTTTGTTAACGACGATGACCAGCTGTATGTTATATACAATCCAC
2792	GGTTCTTGAAGATAAAATTCGTTTGTTCTG
2865	CTCGTCTCCGGTGGAATGA
2866	CGTTCATTCGAAACAAGGAC
2881	CTGGTGAACCGCATCGAGCTGAAG
2882	GAATATACAAATGGGCACACCAATGA
2906	CGAGCAGAAGTTGATAAGCGAA
2908	CTGGTGAACCGCATCGAGCTGA
2909	GTATGTTATATACAATCCACACATCCATTCA
2910	AGTTTGGTGACGATGATAATGCTAAGT
2911	TGGAAAGGCTTCTGCTCAGAGT
2912	ATACTGGACCTAAATCTCCTGAGTTC
2913	CAGAGTTGGTTACGTGAGATTAGAATATG
2946	AAAGGGAAGGAAGTTCAGGGAGCTCGGGCGGCATTGGAGGTCCACACGAA
2947	TATGTGAGCGCCCCTCCCCCCCCAAAATATTTTACACTTTATGCTTCCGG
2969	GAGGACAGACACCAAGACCA
2972	AAGTTGTGTAAAGATACGGTGTTACTG
2973	AAAACAGTAACACCGTATCTTTACACA
3179	GCTTGGGGGGATCCCCGTTGTTCGAGGATTGC
3180	GTTGTGAGTATCTAAATAAAACATAATTAAA
3181	TATTTAGATACTCACAACATATTATATCTTGG
3181	TATTTAGATACTCACAACATATTATATCTTGG
3182	GATGTTCATGAGCCTATAAAGTTGG
3183	TATAGGCTCATGAACATCCAC
3184	GCTGCCATATCCCTCGAGTATATATATATTTTAACTAATAACTTCGT
3192	AGCATTCCAACAAACGGAAG
3203	GAATGTGGATAATTTGGGAACGGA
3396	CGTTGGCAAACCTGGATCCTTTTGGTGGTCGTGGAT
3397	GATCCAGGTTTGCCAACGAATCA
3399	AACTCGCGTCGAACATGTCAGCACTTGCGAGAAAAACGTCA
3400	ACATGTTCGACGCGAGTTCCCT
3401	CTCCTCTCCGTCTGCGCCCTTCT
3402	GGCGCAGACGGAGAGGAGGAAGACGGCGAAGATTCTTCTG
3423	AAAGGGAAGGAAGTTCAGGGAGCTCGGGCGGGCGGCTCTGGAGGCTCAGG
3428	ACAGATACACAGCCACGCATACCTGTAGCGCCCGGGCTGCAGGAATTCAT
3542	TTTCTTGAATTCCCTTTTTCGACAAAATGGC
3543	TCTGTGGGCTGCAGGTTAATCTAGTGGATCCT
3544	CCAGGTTCGTTTGGGGAACTTTT
3545	TTCCCCAAACGAACCTGGTACAACAT

Supplementary Notes

Supplementary Note 1 │ Putative domains of RASP2

A BLAST search of TgRASP2 did not reveal the presence of any putative domains. However, Phyre2[3] and ITASSER[4-6] servers identified a C2 and PH domain with high confidence (98.2 % for C2 and 97.4 % for PH) albeit with poor sequence identity (12 % for C2 and 15 % for PH). Given the evolutionary distances and poor sequence identity of TgRASP2_C2 and PH domains to canonical C2/PH domains, we decided to perform a structure driven sequence alignment using DALi[8] pairwise. We used the top hits predicted by Phyre2 and ITASSER for the comparison. The DALi search revealed statistically significant Z-scores (15.5 for TgRASP2_C2/Rn_C2_Munc13 & 11.4, 22.3 for TgRASP2_PH/Mm_PH/ and TgRASP2_PH/Hs_PH) with low rmsd (1.2 Å over 127 Cα for TgRASP2_C2/Rn_C2_Munc13 and 2.9 Å over 130 Cα for TgRASP2_PH/Mm_PH and 0.2 Å over 117 Cα for TgRASP2_PH/Hs_PH)), suggesting that both TgRASP2_C2 and PH domains may adopt a fold and architecture similar to canonical C2 and PH domains.

Supplementary Note 2 │ Calcium is dispensable for TgRASP2 function.

Many of the proteins possessing a C2 domain require Ca^{2+} for their lipid binding function[9,10]. While the lipid binding property of TgRASP2 is established in our paper (Fig. 1f), we wanted to assess its link to Ca^{2+}.

The capacity for C2 domain-containing proteins is mediated largely by a network of aspartate residues located on a pair of surface loops designated as Loop 1 (L1) and Loop 3 (L3) (Supplementary Fig. 9d). Notably, however, a sequence comparison of Apicomplexan RASP2 C2 homologs with previously published Ca^{2+} dependent and independent C2 containing proteins revealed that most of the key aspartate residues in L1 and L3 have been

replaced by basic amino acids (Supplementary Fig. 9d). These observations suggest a Ca^{2+}-independent function for TgRASP2_C2. To further investigate this observation, we generated a homology model of the TgRASP2_C2 domain. While the model predicted a similar architecture to canonical C2 with a core 8 strand beta sandwich (Supplementary Fig. 3b)[9,10], it also revealed that the surface chemistry of L1 and L3 are unlikely to support Ca_2^+ binding consistent with sequence analysis. Interestingly, the highest scoring template C2 models used for modeling TgRASP2_C2 (PDB: 2CJS & 2CJT) are involved in protein-protein interactions that do not bind Ca^{2+} [11]. Therefore, it is tempting to speculate that TgRASP2_C2 domain might function as a protein-protein interaction module that is independent of Ca^{2+} binding.

Supplementary Note 3 │ The putative PH domain and the three lysine residues of the loop3 in the C2 domain of RASP2 cooperate for rhoptry secretion.

To unravel the function of the putative C2 domain for rhoptry secretion, we first complemented the KD-TgRASP2 mutant with an additional copy truncated for the C2 domain. The ΔC2 construct was completely mistargeted (Supplementary Fig. 10b), therefore precluding any conclusions on the function of this domain for TgRASP2. We then mutated the three lysines of the loop3 in this C2 domain. These were predicted to potentially contribute to lipid binding (Supplementary Fig. 9b). The residues K123 /K128 /K129 were replaced by aspartates and the resulting mutant protein, named L3*, was correctly positioned at the apical end of the tachyzoite. This complementation restored the growth of the KD-TgRASP2 mutant in the presence of ATc, showing that these residues are not essential for the function of the protein. In order to test the function of the putative PH domain and the downstream stretch of polybasic residues (PBR), we generated ΔPH

and ΔPBR constructs. While the ΔPH construct was partially mistargeted, it fully restored the phenotype of TgRASP2 mutant by plaque assay (Supplementary Fig. 10c). The ΔPBR also fully complemented the mutant, showing that individually the domains are dispensable.

Finally, we engineered a combination of double and triple mutations in TgRASP2 and showed an important reduction of lysis plaque size when the lysines within the loop3 of the C2 where combined with a deletion of the PH domain (complementation mutant named ΔPH_L3*). It is worth noting that the localization (Supplementary Fig. 10b) and expression (Supplementary Fig. 10e) did not dramatically differ between the two constructs. We then performed rhoptry secretion assays, and showed a strong defect in rhoptry secretion for the ΔPH_L3* mutant (Fig. 7d). Altogether, this complementary study reveals a cooperative role of the putative PH domain and the negatively charged residues of the loop 3 of the C2 domain.

Supplementary References

1 Besteiro, S., Michelin, A., Poncet, J., Dubremetz, J. F. & Lebrun, M. Export of a *Toxoplasma gondii* rhoptry neck protein complex at the host cell membrane to form the moving junction during invasion. *PLoS Pathog* **5**, e1000309 (2009).

2 Mueller, C. *et al.* The *Toxoplasma* protein ARO mediates the apical positioning of rhoptry organelles, a prerequisite for host cell invasion. *Cell Host Microbe* **13**, 289-301, doi:10.1016/j.chom.2013.02.001 (2013).

3 Kelley, L. A., Mezulis, S., Yates, C. M., Wass, M. N. & Sternberg, M. J. The Phyre2 web portal for protein modeling, prediction and analysis. *Nat Protoc* **10**, 845-858, doi:10.1038/nprot.2015.053 (2015).

4 Roy, A., Kucukural, A. & Zhang, Y. I-TASSER: a unified platform for automated protein structure and function prediction. *Nat Protoc* **5**, 725-738, doi:10.1038/nprot.2010.5 (2010).

5 Yang, J. *et al.* The I-TASSER Suite: protein structure and function prediction. *Nat Methods* **12**, 7-8, doi:10.1038/nmeth.3213 (2015).

6 Zhang, Y. I-TASSER server for protein 3D structure prediction. *BMC bioinformatics* **9**, 40, doi:10.1186/1471-2105-9-40 (2008).

7 Sidik, S. M. *et al.* A Genome-wide CRISPR Screen in Toxoplasma Identifies Essential Apicomplexan Genes. *Cell* **166**, 1423-1435 e1412, doi:10.1016/j.cell.2016.08.019 (2016).

8 Holm, L. & Sander, C. Protein structure comparison by alignment of distance matrices. *J Mol Biol* **233**, 123-138, doi:10.1006/jmbi.1993.1489 (1993).

9 Corbalan-Garcia, S. & Gómez-Fernández, J. C. Signaling through C2 domains: more than one lipid target. *Biochimica Et Biophysica Acta (BBA)-Biomembranes* **1838**, 1536-1547 (2014).

10 Nalefski, E. A. & Falke, J. J. The C2 domain calcium-binding motif: structural and functional diversity. *Protein Science* **5**, 2375-2390 (1996).

11 Lu, J. *et al.* Structural basis for a Munc13–1 homodimer to Munc13–1/RIM heterodimer switch. *PLoS biology* **4**, e192 (2006).
