# Supplementary material for: A lipid-binding protein mediates rhoptry discharge and invasion in Plasmodium falciparum and Toxoplasma gondii parasites
Source: Nat Commun. 2019 Sep 6;10:4041. doi: 10.1038/s41467-019-11979-z (PMC6731292; doi:10.1038/s41467-019-11979-z)
Supplement: Supplementary file 3 — Reporting Summary [file 41467_2019_11979_MOESM3_ESM.pdf]

nature research

Corresponding author(s): Maryse Lebrun

Reporting Summary

Nature Research wishes to improve the reproducibility of the work that we publish. This form provides structure for consistency and transparency in reporting. For further information on Nature Research policies, see Authors & Referees and the Editorial Policy Checklist.

Statistical parameters

When statistical analyses are reported, confirm that the following items are present in the relevant location (e.g. figure legend, table legend, main text, or Methods section).

n/a	Confirmed	
☐	☒	The exact sample size (*n*) for each experimental group/condition, given as a discrete number and unit of measurement
☐	☒	An indication of whether measurements were taken from distinct samples or whether the same sample was measured repeatedly
☐	☒	The statistical test(s) used AND whether they are one- or two-sided<br>*Only common tests should be described solely by name; describe more complex techniques in the Methods section.*
☒	☐	A description of all covariates tested
☒	☐	A description of any assumptions or corrections, such as tests of normality and adjustment for multiple comparisons
☐	☒	A full description of the statistics including central tendency (e.g. means) or other basic estimates (e.g. regression coefficient) AND variation (e.g. standard deviation) or associated estimates of uncertainty (e.g. confidence intervals)
☐	☒	For null hypothesis testing, the test statistic (e.g. *F*, *t*, *r*) with confidence intervals, effect sizes, degrees of freedom and *P* value noted<br>*Give P values as exact values whenever suitable.*
☒	☐	For Bayesian analysis, information on the choice of priors and Markov chain Monte Carlo settings
☒	☐	For hierarchical and complex designs, identification of the appropriate level for tests and full reporting of outcomes
☒	☐	Estimates of effect sizes (e.g. Cohen's *d*, Pearson's *r*), indicating how they were calculated
☐	☒	Clearly defined error bars<br>*State explicitly what error bars represent (e.g. SD, SE, CI)*

Our web collection on statistics for biologists may be useful.

Software and code

Policy information about availability of computer code

Data collection	Gel images were documented using GeneTools analysis software (Syngene). Light and fluorescence microscopy images and time-lapse videos were collected with ZEN (Zeiss). Flow cytometry data was collected using FACS Diva software (BD Biosciences).
Data analysis	All statistical analysis made use of Prism 7 (GraphPad; version 7.0). Light and fluorescence microscopy images, including time-lapse videos, were manipulated using ZEN software (Zeiss; version 2.3 pro). Flow cytometry data was analysed using FACS Diva software (BD Biosciences). Genomic analysis and alignments used BLAST and MUSCLE. Protein structure predictions were performed with Phyre2 and Modeller.

For manuscripts utilizing custom algorithms or software that are central to the research but not yet described in published literature, software must be made available to editors/reviewers upon request. We strongly encourage code deposition in a community repository (e.g. GitHub). See the Nature Research guidelines for submitting code & software for further information.

April 2018

Data

Policy information about availability of data

All manuscripts must include a data availability statement. This statement should provide the following information, where applicable:

- Accession codes, unique identifiers, or web links for publicly available datasets
- A list of figures that have associated raw data
- A description of any restrictions on data availability

The data supporting the results of this study are available within the paper and its Supplementary Information

Field-specific reporting

Please select the best fit for your research. If you are not sure, read the appropriate sections before making your selection.

☒ Life sciences ☐ Behavioural & social sciences ☐ Ecological, evolutionary & environmental sciences

For a reference copy of the document with all sections, see nature.com/authors/policies/ReportingSummary-flat.pdf

Life sciences study design

All studies must disclose on these points even when the disclosure is negative.

Sample size	Sample sizes varied across the study as follows:<br>All genomic analyses (e.g. diagnostic PCR) used samples of DNA purified from at least 10 million T. gondii parasites and P. falciparum parasites (estimate).<br>All Western blot blot analyses used samples of extracts generated from at least 6 million parasites.<br>FACS analyses to determine ring counts measured the numbers of parasite-infected cells in a total of 100,000 red blood cells per timepoint. Since parasitaemia values ranged from 1-18 %, this was considered sufficient to provide statistically robust measurements of ring counts.<br>Other specific values (e.g. number of egress events used to determine egress efficiency) are provided in the figure legends and/or Methods section.
Data exclusions	No data were excluded from the analyses
Replication	All attempts at replication were successful in all experiments. All experiments were repeated at least once (i.e. at least 2 biological replicates), and usually more often, as detailed for individual experiments in the paper (usually provided as an 'n' value).
Randomization	Allocation of sample organisms was completely random. This study was entirely in vitro, using wild-type or transgenic microorganisms (Toxoplasma gondii and Plasmodium falciparum).
Blinding	No blinding was performed.

Reporting for specific materials, systems and methods

Materials & experimental systems

n/a	Involved in the study	
☐	☒	Unique biological materials
☐	☒	Antibodies
☐	☒	Eukaryotic cell lines
☒	☐	Palaeontology
☒	☐	Animals and other organisms
☒	☐	Human research participants

Methods

n/a	Involved in the study	
☒	☐	ChIP-seq
☒	☐	Flow cytometry
☒	☐	MRI-based neuroimaging

Unique biological materials

Policy information about availability of materials

Obtaining unique materials	All unique materials are readily available from the authors.

Antibodies

Antibodies used	reported in Methods section 'reagents and antibodies'
Validation	reported in Methods section 'reagents and antibodies'

Eukaryotic cell lines

Policy information about cell lines

Cell line source(s)	Human foreskin fibroblasts (HFFs) (American Type Culture Collection-CRL 1634) and Vero cells (American Type culture Collection CCL 81).<br>This work used a single Plasmodium falciparum clonal line called p230p DiCre, produced in AA Holder lab and described in Knuepfer et al. (2017) Sci Rep. 2017 Jun 20;7(1):3881<br>PMID: 28634346 (cited in the manuscript).
Authentication	No further authentication was conducted in our laboratory.
Mycoplasma contamination	The cell lines were negative for mycoplasma contamination.
Commonly misidentified lines<br>(See ICLAC register)	No commonly misidentified cell lines were used.
