# Supplementary material for: A lipid-binding protein mediates rhoptry discharge and invasion in Plasmodium falciparum and Toxoplasma gondii parasites
Source: Nat Commun. 2019 Sep 6;10:4041. doi: 10.1038/s41467-019-11979-z (PMC6731292; doi:10.1038/s41467-019-11979-z)
Supplement: Supplementary file 4 — Description of Additional Supplementary Files [file 41467_2019_11979_MOESM4_ESM.pdf]

Description of Additional Supplementary Files

File Name: Supplementary Movie 1
Description: Time lapse video microscopy of merozoites egressing from schizonts in the DMSO control population of iKO-PfPRASP2-HA3. Relative time shown in seconds.

File Name: Supplementary Movie 2
Description: Time lapse video microscopy of PfRASP2-depleted merozoites egressing from schizonts in the rapamycin-treated population of iKO-PfPRASP2-HA3. (= KO) Relative time shown in seconds.

File Name: Supplementary Movie 3
Description: Three-dimensional animation reconstruction from 20 serial SR-SIM sections of the apical region of TgRASP1-HA3 parasites. The inner membrane complex (IMC, blue), the rhoptry neck (RON9, green) and TgRASP1-HA3 (red).
